# Elder abuse within the family environment in the Azores Islands ^1^

**DOI:** 10.1590/1518-8345.1871.2932

**Published:** 2017-09-18

**Authors:** Juan Manuel Carmona-Torres, Rosa María Carvalhal-Silva, Maria Helena Viera-Mendes, Beatriz Recio-Andrade, Thomas Goergen, María Aurora Rodríguez-Borrego

**Affiliations:** 2 PhD, Researcher, Instituto Maimónides de Investigación Biomédica de Córdoba, Universidad de Castilla La Mancha, Toledo, Castilla La Mancha, Spain.; 3 PhD, Professor, Escola Superior de Saúde, Universidade dos Açores, Angra do Heroísmo, Açores, Portugal.; 4 MSc, Adjunct Professor, Escola Superior de Saúde, Universidade dos Açores, Angra do Heroísmo, Açores, Portugal.; 5 RN, Hospital Virgen de la Salud, Toledo, Castilla La Mancha, Spain.; 6 PhD, Adjunct Professor, Deutsche Hochschule der Polizei, Münster, NRW, Germany.; 7 PhD, Full Professor, Instituto Maimónides de Investigación Biomédica de Córdoba, Universidad de Córdoba, Córdoba, Andalucía, Spain.

**Keywords:** Elder Abuse, Aged, Domestic Violence, Azores

## Abstract

**Objectives:**

to dimension abuse against vulnerable adults within the family and community environment in the Azores Islands, identify risk factors for abuse and describe the profile of an abused elder.

**Method:**

descriptive cross-sectional study. Random sampling. The instruments used were: clinical histories of the users, Mini-Mental State Examination, Index of Independence in Basic Activities of Daily Living, Family APGAR Scale, Elder Abuse Suspicion Index and Social Work Assessment Form. Descriptive statistical analysis was used for qualitative and quantitative variables and multiple logistic regression was used to identify factors associated with elder mistreatment.

**Results:**

abuse suspicion was identified in 24.5% of elderly participants. Psychological abuse was the most common type of abuse and sons were the main abusers.

**Conclusion:**

being a woman and belonging to a dysfunctional family is associated with an increased risk of becoming a victim of abuse; the high level of domestic violence against the elderly in the Azores Islands is in line with the rest of Portugal.

## Introduction

Elder abuse was defined in the Toronto Declaration on the Global Prevention of Elder Abuse^1^ as “Single or repeated act, or lack of appropriate action, occurring within any relationship where there is an expectation of trust which causes warm or distress to an older person”. 

Abuse detection is a very complex task because in many cases this problem remains hidden and is concealed by the victims themselves^2^. The low detection of elder abuse has many causes, among which is the fact that it occurs mainly in the private sphere^2^. The victim denies the abuse and does not report it due to a fear of retaliation, guilt, shame, or fear of not being believed; or, on the other hand, because they are affected by cognitive impairment or because they are socially isolated. Another major obstacle is the abuser, who denies abuse, prevents access to health and/or social services, and rejects interventions. In turn, the professionals responsible for care in these situations may lack training, protocols, time and awareness on the issue; or do not want to get involved in legal issues, are unaware of the available resources, are afraid of the anger of the person responsible for the elderly or display a reticent behavior^1^ ^-^ ^3^. 

In Portugal there is no specific legislation to protect groups of vulnerable adults^4^. The existing legislation whereby elder abuse is tried is based on general rules of the criminal procedure code, not exclusive to elderly people, such as article 143 “crimes against physical integrity” or article 152 “crime of domestic violence”^5^. Previous studies on elder abuse in Portugal are scarce^6^ ^-^ ^8^, and although they use different measurement instruments, most identify psychological abuse as the most common type of abuse. The estimated prevalence rates of elder abuse within the family environment in Portugal range from 12.3% to 51.8%^6^ ^-^ ^8^. The World Health Organization (WHO)^7^, as a result of the analyzes of the “European Report on Preventing Elder Maltreatment” (2011), states that “elder abuse is particularly serious in Portugal”. As regards the Azores Islands, recognition of abuse as a public health issue is relatively recent. References in the medical literature goes back over 30 years and it has been an important topic of debate and concern, along with the rapid population aging. A 2009 gender-based violence study in people aged 18 years and over, found that 34.2% of people over 65 had been a victim of violence at least once, with psychological violence being the most common^9^.

Experiences of mistreatment have physical, psychological or social consequences for the elderly^7^ ^,^ ^10^ and have an important impact on the health system, since they cause an increase in the health services visits and hospitalizations and may lead to premature mortality^7^ ^,^ ^10^. Therefore, elder abuse has become a reality that health professionals have to deal more and more frequently. This is particularly important in the case of nursing professionals, since they are the ones who perform most part of the home visits and, at times, they are the only ones who have contact to the abused elderly, playing a key role in the detection of elder abuse^11^ ^-^ ^12^. 

For all these reasons, it was decided to investigate the situation of this social issue in the islands of the Azores archipelago, in order to diagnose the prevalence of abuse against vulnerable adults within the family and community environment, identify risk factors for mistreatment and describe the profile of an abused elder in the Azores Islands.

## Method

Cross-sectional observational descriptive study carried out from January to June 2015. The study subjects were people aged over 65 years, men and women, belonging to the area covered by the Primary Care Centers of the Public Health System of the Azores Islands. The inclusion criterion was that the elderly were under the same form of cohabitation for at least six months and the exclusion criterion was elderly people with cognitive impairment.

Sample size was calculated using the GRANMO program (v 7.12 April 2012) to reach a prevalence rate of 34.2%, an estimate based on a previous study conducted in the Azores Islands^9^. The alpha error was set at 0.95 with an accuracy of +/- 0.07 units in a bilateral hypothesis testing and a 10% replacement index, resulting in a sample composed of 196 subjects. 

The instruments used for data collection were: medical records of the users of reference health centers and a single document, which compiles the following instruments: a) Mini-Mental State Examination (MMSE)^13^ b) Index of Independence in Basic Activities of Daily Living (ADL)^14^; validity ranging from 0.66 to 0.93 and reliability of 0.96. c) Family APGAR Scale (Adaptability, Partnership, Growth, Affection, and Resolve)^15^; validity of 0.8 and reliability of 0.75. d) Elder Abuse Suspicion Index (EASI)^16^; sensitivity ranging from 0.47 to 0.55 and specificity of 95%. e) Social Work Assessment Form (SWAF)^16^, which consists of 67 questions, with question 59 “Do you think this patient has been abused?” considered as an indicative variable to identify if this is a case of elder abuse. In addition, this variable is used to compare and validate EASI results.

The independent variables of this study were: data of the elderly (age, sex, marital status, children, number of children, economic income, level of education, work activity, official minimum income, type of cohabitation, with whom he lives, cohabitation length under the same conditions); data of the cohabiting family members (number of cohabitants, age, sex, marital status, economic income, work activity and official minimum income); evaluation of cognitive impairment; evaluation of the degree of dependence; family functionality and type of abuse suspicion. The dependent variables were: suspicion of abuse (yes/no) and interviewer’s suspicion based on objective data on whether the patient had been abused (yes, no, I do not know).

In order to carry out the study, the corresponding ethics committee’s approval was requested and the health professionals and/or partners of the Azores Islands Health Centers (Nurses and/or Social Workers and/or Psychologists) were contacted. These professionals were the means of access to the study population and the ones who interviewed the participants in the health center or in their home in cases in which patients could not travel due to health problems. All participants received a Study Information Sheet and filled out the Informed Consent Form prior to initiating the interviews. The interview was semi-structured (applying the various questionnaires mentioned above).

Statistical analysis of the data was performed using the PASW Statistic 18 software and consisted of: a) descriptive analysis for qualitative variables, through the calculation of counts (n) and proportions (%), and for quantitative variables, through the calculation of arithmetic mean (m) and standard deviations (SD). b) multivariate association: multinomial logistic regression analysis (MLR) was used to identify possible factors associated with mistreatment (to check the association between independent variables and the abuse suspicion variable). All contrasting hypotheses were bilateral. In all statistical tests, the “significant” values were those whose confidence level was 95% (p<0.05).

This study was conducted in accordance with the fundamental principles of the Declaration of Helsinki and the Portuguese legislation: Law 67/98, of 26 October, on the protection of personal data (transposition to the Portuguese legal system of Directive 95/46/EC of the European Parliament and of the Council of 24 October 1995, on the protection of individuals with regard to the processing of personal data and on the free movement of such data).

## Results

In total, 212 elderly people participated in the study. The sociodemographic characteristics of the elderly people are shown in Table 1, noting that 72.2% were women and 27.8% were men, with an average age of 74.91 (SD±6.859). Since cognitive impairment was an exclusion criterion in the study, all individuals were able to participate in the interview and fill out the EASI. The mean MMSE score was 29.95 points (SD±3.926). With regard to ADL, the mean score was 89.2 points (SD±19.036), so that 50.7% were independent and 24.2% had moderate dependence. Regarding the Family APGAR Scale, the mean score was 8.43 points (SD±2.26), with 84.9% of the elderly people belonging to normofunctional families.

**Table 1:** Sociodemographic characteristics of the elderly (n=212). Azores Islands, PT, Portugal, 2015

**Qualitative variables**		**N**	**%**
**Sex**			
	**Man**	**59**	**27.8**
	**Woman**	**153**	**72.2**
**Marital Status**			
	**Married**	**103**	**48.6**
	**Widowed**	**82**	**38.7**
	**Divorced**	**11**	**5.2**
	**Separated**		
	**Single**	**14**	**6.6**
	**Common-law marriage**	**2**	**0.9**
**Level of Education**			
	**Illiteracy**	**29**	**13.7**
	**Non-formal elementary education**	**31**	**14.6**
	**Incomplete elementary education**	**79**	**37.3**
	**Complete elementary education**	**68**	**32.1**
	High school education/v.t.* incomplete	**1**	**0.5**
	High school education/v.t.* complete	**2**	**0.9**
	**Higher Education**	**2**	**0.9**
**Work activity**			
	**Yes**	**5**	**2.4**
	**No**	**207**	**97.6**
**Economic Income**			
	**Yes**	**160**	**75.5**
	**No**	**52**	**24.5**
**Official Minimum Income**			
	**Yes**	**202**	**95.3**
	**No**	**10**	**4.7**
**Do you have children?**			
	**Yes**	**182**	**85.8**
	**No**	**30**	**14.2**
**Type of cohabitation**			
	**Permanent**	**75**	**33**
	**Intermittent (temporary)**	**137**	**67**
**Form of Cohabitation**			
	**Alone**	**53**	**25**
	**Spouse**	**77**	**36.3**
	**Spouse and children**	**21**	**9.9**
	**Spouse, children and grandchildren**	**5**	**2.4**
	**Spouse and grandchildren**	**3**	**1.4**
	**Spouse and another 1** ^**st**^ **and 2** ^**nd**^ **degree relative**	**0**	
	**Children**	**33**	**15.6**
	**Children and grandchildren**	**8**	**3.8**
	**Grandchildren**	**2**	**0.9**
	**Another 2** ^**nd**^ **and 3** ^**rd**^ **degree relative**	**6**	**2.8**
	**Caregiver**	**4**	**1.9**
	**Another form not specified**	**0**	
**Dependence in ADL** ^**†**^			
	**Total Dependence**	**4**	**1.9**
	**Severe Dependence**	**18**	**8.5**
	**Moderate Dependence**	**51**	**24.2**
	**Mild Dependence**	**31**	**14.7**
	**Independent**	**107**	**50.7**
**Family functionality**			
	**Normofunctional**	**180**	**84.9**
	**Mild dysfunction**	**26**	**12.3**
	**Severe dysfunction**	**6**	**2.8**
**Quantitative variables**		**Mean**	**SD**
	**Age**	**74.9**	**6.9**
	**Children**	**2.7**	**1.9**

*v.t.: vocational training; †ADL: Independence in Basic Activities of Daily Living

With respect to the EASI, which measures the abuse suspicion in the last year, abuse suspicion was identified in 24.5% of the cases. In such cases of abuse suspicion, suspicion of psychological abuse was the most frequent (46.66%), followed by negligence (30%), economic abuse (13.33%) and physical abuse (10%). In these cases, the elderly identified as an abuser his sons (43.45%), spouse (26.09%), daughter-in-law or son-in-law (13.05%), nephews (8.7%), neighbors (8.7%), and in 69.54% of cases the abuser was a first-degree relative. Regarding SWAF, in 9.5% of the cases, the interviewer’s suspicion that the patient had been abused was based on objective data: such as clothing, dirt, bad smell, etc., and the expression of the perception of the elderly person himself. 

As regards the profile of the elderly person in cases of abuse suspicion, Table 2 shows that 65.4% were women and 34.6% were men, with a mean age of 74.04 years (SD±6.556). Most were married (44.2%) and 82.7% had children with an average of 2.35 children (SD±1.877). The most common cohabitation was with the spouse (36.5%). Incomplete elementary education was the most frequent level of education in this group (46.2%). Regarding the work activity, 100% had no work activity and 7.7% did not reach the official minimum income. Approximately one-third of the participants were independent in ADL (36.5%) and belonged to normofunctional families (61.6%).

**Table 2:** Sociodemographic characteristics of elderly people with abuse suspicion and without abuse suspicion. Azores Islands, PT, Portugal, 2015

**Sociodemographic variables of the elderly people**		**Abuse Suspicion**			**No Abuse Suspicion**	
		**N**	**%**		**N**	**%**
**Sex**						
	**Man**	**18**	**34.6**		**41**	**25.6**
	**Woman**	**34**	**65.4**		**119**	**74.4**
**Marital Status**						
	**Married**	**23**	**44.2**		**80**	**50**
	**Widowed**	**20**	**38.5**		**62**	**38.8**
	**Divorced**	**3**	**5.8**		**8**	**5**
	**Separated**	**5**	**9.6**		**9**	**5.6**
	**Common-law marriage**	**1**	**1.9**		**1**	**0.6**
**Level of Education**						
	**Illiteracy**	**7**	**13.5**		**22**	**13.7**
	**Non-formal elementary education**	**5**	**9.6**		**26**	**16.3**
	**Incomplete elementary education**	**24**	**46.2**		**55**	**34.4**
	**Complete elementary education**	**14**	**26.9**		**54**	**33.8**
	High school education/v.t.* incomplete	**0**			**1**	**0.6**
	High school education/v.t.* complete	**1**	**1.9**		**1**	**0.6**
	**Higher Education**	**1**	**1.9**		**1**	**0.6**
**Work Activity**						
	**Yes**				**5**	**3.1**
	**No**	**52**	**100**		**155**	**96.9**
**Economic Income**						
	**Yes**	**35**	**67.3**		**125**	**78.1**
	**No**	**17**	**32.7**		**35**	**21.9**
**Official Minimum Income**						
	**Yes**	**48**	**92.3**		**154**	**96.2**
	**No**	**4**	**7.7**		**6**	**3.8**
**Do you have children?**						
	**Yes**	**43**	**82.7**		**139**	**86.9**
	**No**	**9**	**17.3**		**21**	**13.1**
**Type of Cohabitation**						
	**Permanent**	**16**	**37.2**		**44**	**31.7**
	**Intermittent (temporary)**	**27**	**62.8**		**95**	**68.3**
**Form of Cohabitation**						
	**Alone**	**12**	**23.1**		**41**	**25.6**
	**Spouse**	**19**	**36.5**		**58**	**36.3**
	**Spouse and children**	**6**	**11.6**		**15**	**9.4**
	**Spouse and grandchildren**	**0**			**3**	**1.9**
	**Spouse, children and grandchildren**	**0**			**5**	**3.1**
	**Children**	**11**	**21.2**		**22**	**13.8**
	**Children and grandchildren**	**1**	**1.9**		**7**	**4.4**
	**Grandchildren**	**0**			**2**	**1.2**
	**Another 2** ^**nd**^ **and 3** ^**rd**^ **degree relative**	**1**	**1.9**		**5**	**3.1**
	**Caregiver**	**2**	**3.8**		**2**	**1.2**
**Dependence in ADL** ^**†**^						
	**Total Dependence**	**2**	**3.9**		**2**	**1.3**
	**Severe Dependence**	**8**	**15.4**		**10**	**6.3**
	**Moderate Dependence**	**13**	**25**		**38**	**23.9**
	**Mild Dependence**	**10**	**19.2**		**21**	**13.2**
	**Independent**	**19**	**36.5**		**88**	**55.3**
**Family functionality**						
	**Normofunctional**	**32**	**61.6**		**148**	**92.5**
	**Mild dysfunction**	**15**	**28.8**		**11**	**6.9**
	**Severe dysfunction**	**5**	**9.6**		**1**	**0.6**

*v.t.: vocational training; †ADL: Independence in Basic Activities of Daily Living

Significant differences were found with a value of p=0.011 when comparing the total ADL score, and those with abuse suspicion showed lower ADL test scores (83.37 points, SD±24,569) than those without abuse suspicion (91.09 points, SD±16.509). Significant differences with a value of p<0.001 were also found when comparing abuse suspicion with Family APGAR, with dysfunctional families being more prone to abuse (83.33% of dysfunctional families showed abuse suspicion).

The odds ratios (OR) resulting from multiple logistic regression analyses are shown in Table 3. These results confirm that elderly people belonging to a family with mild or severe dysfunction are 8.351 times more prone to have abuse suspicion than those belonging to a normofunctional family (95%CI: 3.647-19.122), and women are 1.871 times more prone to abuse suspicion than men (95%CI: 0.901-3.887).

**Table 3:** Factors associated with elder abuse, according to multiple logistic regression analyses. Azores Islands, PT, Portugal, 2015

		**p-value**	**Odds Ratio**	*95%CI
**Family functionality**				
	**Normofunctional**	**-**	**1**	**-**
	**Mild or severe dysfunction**	**<0.001**	**8.351**	**3.647-19.122**
**Sex**				
	**Man**	**-**	**1**	**-**
	**Woman**	**0.093**	**1.871**	**0.901-3.887**

*Confidence interval

## Discussion

Based on these results, it can be said that gender influences the probability of an elderly person being abused. These results are consistent with the existing literature on this subject^17^ ^-^ ^19^, such as a study conducted in the province of Malaga (Spain), in which of the 259 cases of elder abuse analyzed, 77.6% of the victims were women^18^. Other studies add that the greater the age, the greater the vulnerability^6^, so that age is considered another risk factor for mistreatment. Dependence in ADL is also related to being a victim of abuse, as people with abuse suspicion have low scores on the ADL test. This is consistent with other studies^6^ ^,^ ^17^ ^-^ ^18^, such as one conducted in Spain in which it was found that the abuse rate increases by up to 2.9% among elderly people with severe dependence^17^. Finally, it seems that the functioning of the family structure influences the abuse, so that belonging to a dysfunctional family increases the probability of abuse, as seen in previous studies carried out in other countries^19^. 

Regarding the profile of the abused elderly person in the Azores Islands, this is a married woman with 74 years of age, incomplete elementary education, no work activity and an average of 2.35 children. This profile is similar to that found by most studies conducted in other countries^17^ ^,^ ^19^ ^-^ ^20^, so it seems that there is a unique profile of the abused elderly person, prevailing over cultural and geographical diversity. 

The prevalence of elder abuse found in this study varies in relation to other studies conducted in Portugal, being lower than that of a study where the prevalence was 51.8%^8^ and higher than that of another study in which 12.3% of the elderly people had been abused in the last year^6^. However, these numbers are slightly higher than those found in other European studies, such as Spain, where, according to a study, 12.1% of the elderly people are abused^20^; or Italy, where, according to another study, 12.7% of the elderly people are abused^21^. Compared with the rest of the world, these numbers are lower than those found in American countries, such as Brazil, where, according to a recent study, the prevalence of abuse is 78.1%^22^, or Bolivia, where the prevalence is 39%^19^; or Asia, such as a study conducted in China that found a prevalence of abuse of 36.2%^23^. The reasons explaining the differences in prevalence among countries include the different instruments used to measure abuse.

As in previous studies, psychological abuse is the most common form of mistreatment, followed by negligence^6^ ^,^ ^19^ ^-^ ^21^. In addition, most of the elder abuse is committed by the sons, followed by the partner or spouse, corroborating the existing studies^10^ ^,^ ^17^ ^,^ ^19^ ^-^ ^20^. The fact that a vast majority of the abusers belong to the family nucleus makes it difficult to identify cases of abuse^24^, making these data even more alarming. Since the health professionals are the only ones who, in most cases, have access to the abused elderly person, and nurses are the ones who perform most part of home visits^11^ ^-^ ^12^, it is their responsibility to detect and report cases of elder abuse. Therefore, primary care plays a decisive and fundamental role^2^ ^,^ ^18^ ^,^ ^25^. 

However, some studies conclude that the phenomenon of abuse may go unnoticed due to the lack of training of the health professionals who care for possible victims^26^. In fact, some studies report that nursing students have a lack of training regarding other forms of abuse such as partner violence^27^. Therefore, it would be useful to introduce specific contents on domestic violence into the Nursing undergraduate program^27^. For all these reasons, it seems necessary to implement training programs on elder abuse within the family and community environment, in order to help health professionals to prevent, detect, evaluate and intervene in such an issue^2^ ^,^ ^25^. Several studies show that in the fields where professionals are better trained and motivated, the detection of elder abuse is also better^11^. 

From the results of the medical records analysis, it is also observed that health professionals do not fill out the medical records. The same was observed in the results of other investigations that concluded that, in general, the actual activity is not reflected, there is no uniformity in the records and sometimes there is no connection between the specialized medical care centers^12^. However, the medical record is a fundamental tool to ensure communication between the different members of the multidisciplinary staff^11^. 

Among the limitations of this study, it is observed that the exclusion of elderly people with cognitive impairment from this study is one of the main limitations of it, because according to the literature, the prevalence of elder abuse among elderly people with cognitive impairment is higher than that observed in mentally intact individuals. Another limitation is that EASI has not been developed to identify cases of abuse, but to identify cases of “abuse suspicion”, a limitation that has been mitigated by the application of FETS, by contrasting both results. The same results have been obtained in both questionnaires for cases of abuse, so that both are capable of confirming cases of abuse. Another limitation with respect to the cross-sectional design of the study is that it was not possible to know the direction of causality between abuse and the associated variables.

## Conclusion

The prevalence of elder abuse in the Azores Islands was dimensioned and the profile of the abused elderly has been obtained. The most frequent form of abuse is the psychological one and the sons are the main abusers. Being a woman and belonging to a dysfunctional family are factors associated with an increased probability of being victim of abuse. As requirement for nursing practice, it is worth emphasizing that primary care nursing professionals should aim to detect this problem, by receiving specific training so that the phenomenon does not go unnoticed. In addition, these professionals should work in a comprehensive and interdisciplinary way with the rest of the staff in order to implement tools, programs and protocols to ensure the prevention, detection and intervention in these situations.

## References

[1] WHO (World Health Organizations), INPEA (International Network for the
Prevention of Elder Abuse) (2002). Missing voices. Views of Older Persons on Elder Abuse.

[2] Moya-Bernal A, Barbero-Gutiérrez J, Barrio-Cantalejo IM, Gutiérrez-González B, Fernández de Trocóniz MI, Martínez-Maroto A (2005). Malos tratos a personas mayores: Guía de Actuación [Internet].

[3] Ruelas-González MG, Pelcastre-Villafuerte BE, Reyes-Morales H (2014). Maltrato institucional hacia el adulto mayor: percepciones del
prestador de servicios de salud y de los ancianos. Salud pública de México.

[4] de Magalhães AS (2015). Violence and crimes against the elderly: a Portuguese-Brazilian
perspective. Cad Dereito Actual.

[5] Fonseca R, Gomes I, Faria PL, Gil AP (2012). Perspetivas atuais sobre a proteção jurídica da pessoa idosa vítima de
violência familiar: contributo para uma investigação em saúde
pública. Rev Port de Saúde Pública.

[6] Martins-Gil AP, Kislaya I, Santos AJ, Nunes B, Nicolau R, Fernandes AA (2015). Elder abuse in portugal: findings from the first national prevalence
study. J Elder Abuse & Neglect.

[7] Sethi D, Wood S, Mitis F, Bellis M, Penhale B, Marmolejo II (2011). European report on preventing elder maltreatment [Internet].

[8] Eslami B, Viitasara E, Macassa G, Melchiorre MG, Lindert J, Stankunas M (2016). The prevalence of lifetime abuse among older adults in seven European
countries. Int J Public Health.

[9] Lisboa M, Miguens F, Cerejo D, Favita A (2009). Inquérito violência de género. Região Autónoma dos Açores. Relatório final
[Internet].

[10] Fernández-Alonso MC, Herrero-Velázquez S (2006). Maltrato en el anciano. Posibilidades de intervención desde la
atención primaria (II). Atención Primaria.

[11] Espinosa Monzada C (2009). ¿Asisto a un anciano maltratado. El Peu.

[12] Dios-Guerra C, Carmona-Torres JM, Ruíz-Gándara Á, Muñoz-Alonso A, Rodríguez-Borrego MA (2015). Programmed home visits by nursing professionals to older adults:
prevention or treatment?. Rev. Latino-Am. Enfermagem.

[13] Brito-Marques PR, Cabral-Filho JE (2005). Influence of age and scholing on the performance in a modified
Mini-Mental State Examination version: a study in Brazil Northeast. Arq Neuro-psiquiatria.

[14] Araújo F, Ribeiro JLP, Oliveira A, Pinto C (2007). Validação do Índice de Barthel numa amostra de idosos não
institucionalizados. Rev Portuguesa Saúde Pública.

[15] Agostinho M, Rebelo L (1988). Família: do conceito aos meios de comunicação. Rev Portuguesa de Saúde Pública.

[16] Yaffe MJ, Wolfson C, Lithwick M, Weiss D (2008). Development and validation of a tool to improve physician
identification of elder abuse: The Elder Abuse Suspicion Index
(EASI)©. J Elder Abuse & Neglect.

[17] Iborra I (2008). Maltrato de personas mayores en la familia en España [Elder abuse in the
family in Spain] [Internet].

[18] Castilla-Mora R, Palma-García MO (2014). El maltrato a personas mayores en el ámbito familiar. Aproximación a
la situación en Málaga (España). Revista de investigaciones en intervención social.

[19] Carmona-Torres JM, López-Soto PJ, Coimbra-Roca AI, Gálvez-Rioja RM, Goergen T, Rodríguez-Borrego MA (2015). Elder Abuse in a Developing Area in Bolivia. J Interpersonal Violence.

[20] Pérez-Rojo G, Izal M, Montorio I, Regato P, Espinosa JM (2013). Prevalencia de malos tratos hacia personas mayores que viven en la
comunidad en España. Med Clin (Barc).

[21] Fraga S, Lindert J, Barros H, Torres-Gonzalez F, Ioannidi-Kapolou E, Melchiorre MG (2014). Elder abuse and socioeconomic inequalities: A multilevel study in 7
European countries. Preventive Med.

[22] Irigaray TQ, Esteves CS, Pacheco JTB, Grassi-Oliveira R, Argimon IIDL (2016). Elder abuse in Porto Alegre, Rio Grande do Sul, Brazil: a documentary
study. Estudos de Psicologia (Campinas).

[23] Wu L, Chen H, Hu Y, Xiang H, Yu X, Zhang T (2012). Prevalence and associated factors of elder mistreatment in a rural
community in People’s Republic of China: a cross-sectional study. PloS one.

[24] Risco-Romero C, MdC Paniagua Vicioso, Jiménez-Mendoza G, Poblador-Curtó MD, Molina-Martínez L, Buitrago F (2005). Prevalencia y factores de riesgo de sospecha de maltrato en población
anciana. Med Clin (Barc).

[25] Torres-Prados M, Estrella-González IM (2015). Sensibilización y detección del maltrato en el anciano: hacia una
atención primaria adaptada a los mayores. Gerokomos.

[26] Fonseca-Machado MO, Monteiro JC, Haas Vanderlei J, Abrão AC, Gomes-Sponholz F (2015). Intimate partner violence and anxiety disorders in pregnancy: the
importance of vocational training of the nursing staff in facing
them. Rev. Latino-Am. Enfermagem.

[27] Rigol-Cuadra A, Galbany-Estragué P, Fuentes-Pumarola C, Burjales-Martí MD, Rodríguez-Martín D, Ballester-Ferrando D (2015). Perception of nursing students about couples’ violence: knowledge,
beliefs and professional role. Rev. Latino-Am. Enfermagem.

